# Inter-center variability of dosimetry methodology and the impact on reported absorbed dose for [^177^Lu]Lu-PSMA-617 therapy

**DOI:** 10.1007/s00259-026-07832-3

**Published:** 2026-03-26

**Authors:** B. J. R. Timmermans, B. M. Privé, R. Hofferber, M. W. Konijnenberg, G. Flux, S. Heskamp, J. Nagarajah, J. I. Gear, W. Jentzen, S. M. B. Peters

**Affiliations:** 1 Department of Medical Imaging – Nuclear Medicine, Radboud university medical center, Nijmegen, The Netherlands; 2 Department of Radiation Oncology, Erasmus MC, Rotterdam, The Netherlands; 3 Department of Nuclear Medicine, University Hospital Essen, University of Duisburg- Essen, 45147 Essen, Germany; 4 Department of Nuclear Medicine, Erasmus MC, Rotterdam, The Netherlands; 5 Joint Department of Physics, Royal Marsden Hospital and Institute of Cancer Research, London, UK

**Keywords:** Dosimetry, Variability, [^177^Lu]Lu-PSMA, Radionuclide therapy, Prostate cancer

## Abstract

**Purpose:**

[^177^Lu]Lu-PSMA-617 radioligand therapy improves overall survival in advanced prostate cancer and is under evaluation in early-stage disease. Dosimetry can guide personalized [^177^Lu]Lu-PSMA-617 therapy by determining tumor and organ absorbed doses (ADs), but workflow variability and lack of standardization limit clinical use. To identify factors most influencing AD, we evaluated inter-center variability using a single-center dataset analyzed by three centers, focusing on the effects of volume delineation and time-activity integration.

**Methods:**

Dosimetry was performed at three experienced centers using local workflows. Inter-center comparisons were conducted in three steps: (1) comparing absolute ADs, (2) comparing inter-center variability using the coefficient of variation (CV) per patient and anatomical region, and (3) evaluating the methodological steps by homogenizing data across centers. Homogenizations included organ and tumor volumes for S-factors (volume-homogenization effect), activity within volumes of interest (activity-homogenization effect), and time-activity curve fitting (TAC-fitting effect), allowing assessment of the impact of individual methodological AD differences.

**Results:**

Significant inter-center differences in median ADs were observed for salivary glands (*p* = 0.010), kidneys (*p* = 0.002), liver (*p* = 0.001), and lesions (*p* = 0.001). Variability was highest for the lesions (median CV 71.5%). In organs, variability was highest in salivary glands (median CV 35.7%), followed by liver (median CV 21.2%) and kidneys (median CV 16.8%). Volume-homogenization had the biggest impact on inter-center variability for lesions, reducing median CV to 32.3%; this reduction in variability was less pronounced for organs. Activity-homogenization proved more influential for organs, reducing median CV in salivary glands, kidneys and liver to 12.9%, 11.8% and 11.5%, respectively. The TAC-fitting effect was limited.

**Conclusion:**

Substantial inter-center variability in AD was observed, this was primarily reduced by volume- and activity-homogenization, emphasizing the need for standardized delineation protocols and consensus guidelines to ensure reproducible and comparable dosimetry results.

**Supplementary Information:**

The online version contains supplementary material available at 10.1007/s00259-026-07832-3.

## Introduction

 Radioligand therapy (RLT) has emerged as a promising treatment for prostate cancer (PCa). Lutetium-177 (^177^Lu)-labeled prostate-specific membrane antigen (PSMA) ligands have shown improved overall survival in metastatic castration resistant prostate cancer (mCRPC) [1]. Following the approval of [^177^Lu]Lu-PSMA-617 by the U.S. Food and Drug administration and the European Medicines Agency for use in mCRPC patients that underwent prior chemotherapy, the PSMAfore trial (NCT04689828) recently showed that [^177^Lu]Lu-PSMA-617 also improved progression-free survival in chemotherapy-naïve patients [2]. Ongoing trials are investigating the use of [^177^Lu]Lu-PSMA-617 in earlier stages of PCa, including hormone-sensitive metastatic prostate cancer (mHSPC) [3, 4] and treatment-naive localized PCa [5, 6]. Additionally, extended treatment regimens (e.g. NCT06531499, 12 cycles of 7.4 GBq), alternative PSMA targeting agents (i.e., PSMA-I&T, rhPSMA-10.1, PSMA-R2, J591) and different radionuclides (i.e., actinium-225, terbium-161, astatine-221, lead-212) are investigated for potential therapeutic benefits.

With multiple targeted radiopharmaceuticals emerging, and PSMA-RLT moving to earlier disease settings, the need to evaluate and compare efficacy and safety profiles becomes increasingly important. These patients can have a significantly longer survival, making prevention of potential late toxicity effects more relevant. In addition, a multitude of available treatment options at these disease stages warrants a more detailed assessment of the expected efficacy and toxicity for the chosen therapy. Different PSMA compounds have shown different efficacy and safety profiles. For example, there have been concerns about potential long-term nephrotoxicity after [^177^Lu]Lu-PSMA-I&T [7] as this ligand is suspected to have a higher renal absorbed (radiation) dose (AD) than PSMA-617 [8].

Dosimetry can aid in the assessment of treatment efficacy and toxicity of different [^177^Lu]Lu-PSMA ligands by determining the AD to tumors and organs. Moreover, dosimetry is essential to establish AD-effect relationships. This knowledge can facilitate the personalization of RLT to improve treatment outcome, for example, by optimizing the injected therapeutic activity per cycle, and number and timing of cycles. Personalized radionuclide therapy has proven beneficial in other diseases, such as in hepatocellular carcinoma treated with radioembolization [9] and in neuroendocrine tumors treated with [^177^Lu]Lu-DOTATATE [10]. While this highlights the potential of AD-guided approaches, dosimetry in RLT is no common practice. This is due to multiple factors, such as the lack of established AD-effect relationships and limited availability of dosimetry infrastructure (e.g., SPECT/CT imaging), expertise, and personnel resources. Another paramount factor is the absence of standardization in dosimetry methodologies. Dosimetry involves many steps that can influence the AD calculation. First, the input data for dosimetry can be very different because of differences in imaging time points, the measurement of injected therapeutic activity, type of scanners, acquisition parameters, image reconstruction protocols, equipment calibration and image quantification methods. Second, the dosimetry workflow to determine AD varies largely between institutes due to methodological differences such as organ- vs. voxel-level dosimetry, region drawing, time-activity curve fitting methods, the use of single (first) cycle vs. multi-cycle data, and the choice of imaging modality (planar imaging, SPECT/CT imaging, hybrid approaches). While regulatory frameworks in the United States and Europe require evaluations of the AD to organs-at-risk and tumors [11, 12], they do not specify which methodologies should be used. Overall, this lack of standardization in dosimetry results in significant variability in reported AD and challenges interpretation of results and comparisons within and between research institutes/trials.

To understand which factors in the dosimetry workflow have the biggest impact on calculated AD, we evaluated variations in AD using a dataset collected in a single center, analyzed by three different centers experienced in RLT dosimetry. Comparing AD using one single dataset is ideal to investigate the variability resulting from the dosimetry workflow, as it ensures consistency in imaging methodology, data acquisition and image reconstruction parameters. This way, differences in the reported AD arise solely from the workflow itself. We evaluated the contribution of following factors to the inter-center AD variability in tumors and relevant organs: volume of interest (VOI) delineation, S-factor determination and the remaining variability due to time-activity integration.

## Materials and methods

### Patient population

The data set comprised imaging data of 10 patients with oligometastatic hormone-sensitive prostate cancer (omHSPC) who received [^177^Lu]Lu-PSMA-617 therapy at Radboudumc (Nijmegen, The Netherlands). The original prospective study was approved by the Medical Review Ethics Committee Region Arnhem-Nijmegen and was registered on clinicaltrials.gov (NCT03828838). All patients provided informed consent. Detailed information regarding patient population and clinical outcomes have been previously published [3]. Briefly, patients with omHSPC were included if they had a prostate-specific antigen (PSA) doubling time of ≤ 6 months and ≤ 10 visible metastases on baseline [^68^Ga]Ga-PSMA-11 PET/CT, with at least one lesion ≥ 10 mm in diameter. Only data from the first cycle of [^177^Lu]Lu-PSMA-617 therapy (mean ± standard deviation injected therapeutic activity of 3.1 ± 0.1 GBq) were included in the analysis.

### Image acquisition

Approximately one week prior to RLT, patients underwent [^68^Ga]Ga-PSMA-11 PET/CT imaging. Imaging was performed from vertex to upper thigh on a Biograph mCT system (Siemens Healthineers, Erlangen; Germany) 60 ± 10 min post-injection, following local clinical acquisition protocols (Supplementary Materials S1).

Intra-therapeutic SPECT/CT imaging was performed at 1, 24, 48, 72 and 168 h post-injection of [^177^Lu]Lu-PSMA-617 on either a Symbia T16 or a Symbia Intevo Bold system (Siemens Healthineers, Erlangen; Germany). Both systems were cross-calibrated for ^177^Lu using the in-house dose calibrator. SPECT/CT scans were acquired to include all relevant organs and tumors using three-bed positions: the pelvis, abdomen, and head-neck regions. Detailed acquisition and image reconstruction parameters can be found in Supplementary Materials S1.

### Dosimetry methodology

Three centers were included in the dosimetry comparison, who have experience in performing dosimetry: Radboudumc (Nijmegen, Netherlands), Royal Marsden (London, United Kingdom), and Universitätklinikum Essen (Essen, Germany), referred to as center 1, 2 and, 3, respectively. The ADs were calculated according to methodologies applied clinically in each of the three centers. Centers 1 and 3 collaborated in determining lesion volumes, resulting in identical lesion volumes for both centers. In general, the AD to the tumors and to organs (including kidneys, liver and salivary glands) were determined using volumetric organ-based dosimetry according to the Medical Internal Radiation Dose formulism based on five-time point SPECT imaging. Each center determined a calibration factor for the SPECT/CT data based on provided phantom data from a cylindrical phantom with a volume of 6283 ml: 10.63 cps/MBq (center 1), 10.46 cps/MBq (center 2), 10.31 cps/MBq (center 3). Furthermore, previously identified PCa lesions on [^68^Ga]Ga-PSMA-11 PET/CT [13] were evaluated for dosimetry eligibility by each center independently on pre- and post-treatment imaging. The center-specific dosimetry workflow is described below and summarized in Table 1.

**Table 1:** Overview of key dosimetry steps for each organ/lesion and center

Dosimetry step	Structure	Center 1	Center 2	Center 3
Organ and tumor volumes	Kidneys	Standard masses based on the ICRP89 adult male human model.	CT imaging based.	CT imaging based.
	Liver			
	Salivary glands			CT imaging preferred, iterative PET-based thresholding in case of dental artifacts.
	Lesions	Collaboration with center 3: Based on CT imaging preferred, iterative PET-based thresholding if not possible on CT.	CT imaging based.	Collaboration with center 1: Based on CT imaging preferred, iterative PET-based thresholding if not possible on CT.
Activity determination	Kidneys	VOI using CT-based contouring.	CT-based contouring	VOI based on CT contour in combination with a recovery-coefficient based approach for partial volume and background correction.
	Liver		Three representative smaller VOIs extrapolated to the total liver volume.	
	Salivary glands	Oversized VOI of CT-contour plus approximately 1 cm with background correction using a volume close to the organ.	SPECT/CT based contouring encompassing all counts originating from volume and background correction by extrapolating to a zero-volume count-rate described by Carnegie-Peak et al. [19]	
	Lesions	Oversized VOI of approximately 20–30% larger than the tumor on SPECT/CT was used with background correction using a volume close to the lesion.		Oversized VOI (approximately 1 cm away from the lesion VOI margin) based on the SPECT/CT signal with background correction using a volume close to the lesion.
Time-activity integration	Kidneys	Fitting using Hermes	Mono-exponential curve assuming instantaneous uptake.	Mono-exponential-base trapezoid method.
	Liver			
	Salivary glands			
	Lesions	Mono-exponential curve if R^2^ > 0.7 otherwise trapezoid method.		
AD calculation	Kidneys	ICRP89 reference values	Mass-adjusted S-values	Mass-adjusted S-values.
	Liver			
	Salivary glands			S-values of corresponding sphere volumes of water
	Lesions	S-values of corresponding sphere volumes of water	S-values of corresponding sphere volumes of water	

CT = Computed Tomography; ICRP89 = International commission in radiological protection publication 89; PET = Positron emission tomography; SPECT = Single-Photon Emission Computed Tomography; VOI = Volume of interest

#### Center 1

Detailed methodology has been described previously along with the corresponding results [13]. In the following, a short overview is given.

##### Organ and tumor volumes

For organs, standard masses were used based on the International Commission on Radiological Protection Publication 89 (ICRP89) adult male human model [14]. For tumors, lesion masses were determined on [^68^Ga]Ga-PSMA-11-PET/CT acquired 1 week prior to therapy, preferably using CT imaging, otherwise using a PET-based iterative thresholding method if not clearly visible in CT imaging [15].

##### Activity determination

To determine activity in tumor and organs, VOIs were drawn for each time point. For the liver and kidneys, a contour was drawn based on CT contours of the respective organ. For the salivary glands and tumor lesions, an oversized VOI-based approach was applied to account for partial volume effect. For the salivary gland, the oversized VOI was the organ contour on CT plus a margin of approximately 1 cm. For tumor lesions, an oversized VOI of approximately 20–30% larger than the tumor on SPECT/CT was used. In the oversized-VOI approach, a background correction was applied by drawing a VOI near the oversized VOI and subtracting the relative background counts [16, 17].

##### Time-activity integration

For organs, the time-activity curves (TACs) were constructed in Hermes Dosimetry (Hermes Medical Solutions, Stockholm; Sweden), using either mono-exponential or bi-exponential fitting. For tumor lesions, the trapezoidal method was used between 0 and the first measurement timepoint. For later timepoints, a mono-exponential model was used if the correlation coefficient was above 0.7, otherwise the trapezoid method was used between all timepoints [18]. In case the trapezoidal method was used, the data were extrapolated using the effective half-life between the last two measured timepoints.

##### AD calculation

For organs, S-factors were determined based on the ICRP89 adult male human without mass-scaling. For tumor lesions, S-factors were based on mass-dependent S-factors of corresponding volumes of water (IDAC Dose 2.1). A tissue density of 1 g/ml was assumed to calculate respective masses from the volumes.

#### Center 2

##### Organ and tumor volumes

Organ and lesions volumes were determined based on CT imaging prior to therapy.

##### Activity determination

For the organs, VOIs were drawn for each timepoint based on the anatomical contours of the organs on CT. Three delineation approaches were used. For structures smaller than 10 ml (e.g. lesions and salivary glands), a VOI was drawn encompassing all counts originating from the volume based on the SPECT/CT signal. Two additional VOIs of varying sizes were drawn to enable background correction by extrapolating to a zero-volume count rate [19]. For volumes larger than 10 ml with heterogeneous uptake (e.g. kidneys) a VOI was drawn based on the contour of the volume on CT. For very large structures with homogeneous uptake (e.g. the liver), three small VOIs were used so that uniformity of the object could be ascertained, the average concentration of the spheres were then scaled by the total organ volume to obtain the total organ activity. This small-VOI approach was not applied to the kidneys as even small and circular VOIs within the kidneys remain subject to partial-volume effects [20].

##### Time-activity integration

All TAC curves were created by fitting a mono-exponential curve and assuming instantaneous uptake. Occasionally timepoints were excluded if they did not obviously fit an exponential curve (i.e. the first time point demonstrating continued uptake) or the data points had excessively large activity uncertainty, with uncertainties estimated according to EANM guidelines [21].

##### AD calculation

For liver and kidneys, S-factors were determined by scaling the organ S-factors within OLINDA/EXM 1.1 (Vanderbilt University, Nashville, TN, USA) [22] with the mass determined from the patient CT, assuming a tissue density of 1.05 and 1.06 respectively. For tumor lesions and salivary glands, S-factors were scaled according to mass using unit density sphere models within Olinda.

#### Center 3

The methodology used by center 3 has been described previously [23]. In the following, a short overview is given.

##### Organ and tumor volumes

The volumes for the organs and lesions were determined using [^68^Ga]Ga-PSMA-11-PET/CT acquired 1 week prior to therapy, preferably using CT imaging, otherwise using an iterative PET-based thresholding method if not clearly visible in CT imaging [15].

##### Activity determination

The imaged (average) activity in the organs was derived from the segmented volumes. To account for partial volume effects and for background spill-in, a recovery-coefficient based approach described by Stebner et al. was used [17]. This method conceptualizes the object and surrounding background as two compartments and estimates the spill-in fraction based on the measured activity concentrations in both regions. The delineated activity concentration was corrected by dividing it by a background-specific recovery coefficient. The corrected activity concentration was subsequently used. For the lesions, oversized VOIs (approximately 1 cm away from the segmented VOI margin) were drawn based on the SPECT/CT signal for each timepoint and a respective background VOI was drawn close to the lesions to correct for background as described in literature [16, 17].

##### Time-activity integration

For fitting the TAC for both organs and lesions, a mono-exponential-based trapezoid method was used. First, the trapezoidal method between 0 and the first measurement time point (1 h) was used. Between all other time points a mono-exponential was fitted between each two consecutive timepoints (mono-exponential-based trapezoid method). The last timepoint was extrapolated to infinity using the effective half-life between the last two measured timepoints.

##### AD calculation

S-factors for the organs were mass-adjusted to reflect the specific organ mass. For tumor lesions and for each individual salivary gland types, S-factors were based on S-factors of corresponding sphere volumes. Organ and sphere S-factors are based on OLINDA/EXM 1.1 (Vanderbilt University, Nashville, TN, USA) [22] using a tissue density of 1 g/ml.

### Method of comparison

The comparison between centers consisted of three parts. First, selected organs (kidneys, salivary glands and liver) from all patients (*n* = 10) were included, while only tumors (*n* = 21) for which all centers performed dosimetry were included in the comparison. The reported ADs were compared between centers, comparing both the AD for the whole population across centers and on a patient-level across centers. Second, to compare differences in AD between centers, the coefficient of variation (CV), defined as the standard deviation (SD) expressed as a percentage of the mean, was used. The CV was determined for each anatomical region on a per-patient level and the median and range of CV values across all patients were reported per region. The interquartile range (IQR) and number of outliers were also used to compare the differences in variability across patients. Third, to compare individual steps in the dosimetry methodology, part of the data was homogenized across centers and reanalyzed using the CV to assess the impact on AD. First, organ and tumor volumes used to determine S-factors were homogenized (volume-homogenization effect), second activity determined from the VOI was homogenized (activity- homogenization effect), and thirdly, the first and second homogenization were combined to determine the effect of TAC fitting methods (TAC-fitting effect). All homogenizations were performed based on the volumes and activities determined by center 3, while keeping other methodology used by each center consistent.

### Statistical analysis

Descriptive statistics comprised the median and range. The change in median CV between centers was compared to determine the impact on AD variability at each step. Spearman correlations were used to determine correlation between volume and CV per region. Overall differences between centers were evaluated using the Friedman rank-sum test using the coin package [24] in R version 4.5.2 (R Core Team). When significant, pairwise post-hoc comparisons were performed with a permutation-based nonparametric test for paired differences within the coin package [24] in R version 4.5.2 (R Core Team), with p-values adjusted for multiple comparisons. A p-value ≤ 0.05 was considered statistically significant.

## Results

### Description of the dataset

A total of ten patients were included in the analysis. Dosimetry was performed on all selected organs (liver, kidneys and salivary glands). The number of tumors per patient ranged from one to nine. Each center individually assessed lesion eligibility for dosimetry, and only those deemed eligible by all centers were included in the analysis (19, of which 11 nodal and 8 bone lesions). The primary reason for lesion exclusion was due to limited visibility on the SPECT/CT scan (Supplementary Table 1).

### Variability in ADs between centers

**Fig. 1:**
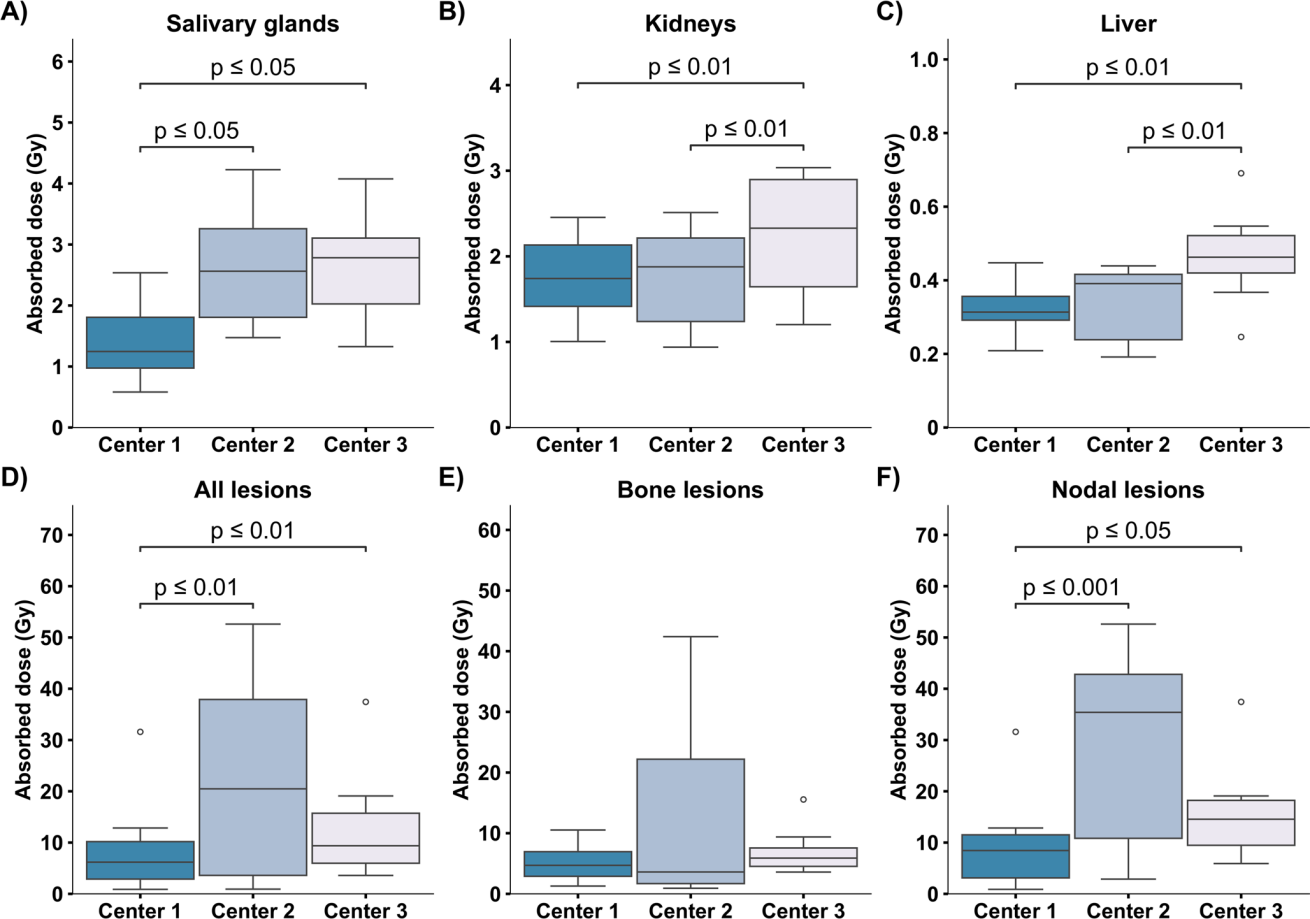
Boxplot of absorbed dose in Gray (Gy) for each center for (**A**) salivary glands, (**B**) kidneys, (**C**) liver, (**D**) lesions, (**E**) bone lesions only, and (**F**) nodal lesions only. Significant post-hoc pairwise differences are indicated on the plots

The absolute ADs determined by each center for organs and tumors are summarized in Fig. 1. Overall, significant differences in median ADs for the salivary glands between centers were observed (*p* = 0.010). Post-hoc pairwise comparisons indicated that center 1 had significantly lower median ADs than center 2 (*p* = 0.024) and center 3 (*p* = 0.024). Similarly, the kidneys and liver showed significant inter-center differences in median AD (*p* = 0.002 and *p* = 0.001, respectively). In these organs, center 3 reported significantly higher median ADs than centers 1 and 2 (kidneys: *p* = 0.002 and *p* = 0.002; liver: *p* = 0.005 and *p* = 0.001, respectively). The median ADs of all lesions differed significantly across centers (*p* = 0.001), with center 1 reporting lower median AD doses than center 2 (*p* = 0.001) and center 3 (*p* = 0.001). When nodal lesions were analyzed separately, similar inter-center differences were observed (*p* = 0.001), again with lower median ADs at center 1 compared with center 2 (*p* = 0.001) and center 3 (*p* = 0.015). In contrast, no significant inter-center differences were found for bone lesions.

For each organ (e.g., salivary glands, kidneys, liver), the maximum difference per patient between the ADs reported by the three centers was calculated. For lesions, the same calculation was performed on a per lesion basis. Across all salivary glands, the median of these maximum differences was 0.39 Gy/GBq (range: 0.14–0.97 Gy/GBq), for the kidneys 0.20 Gy/GBq (range: 0.07–0.40 Gy/GBq), for the liver 0.05 Gy/GBq (range: 0.01–0.12 Gy/GBq), and across all lesions 4.53 Gy/GBq (range: 0.30–14.89 Gy/GBq).

**Fig. 2:**
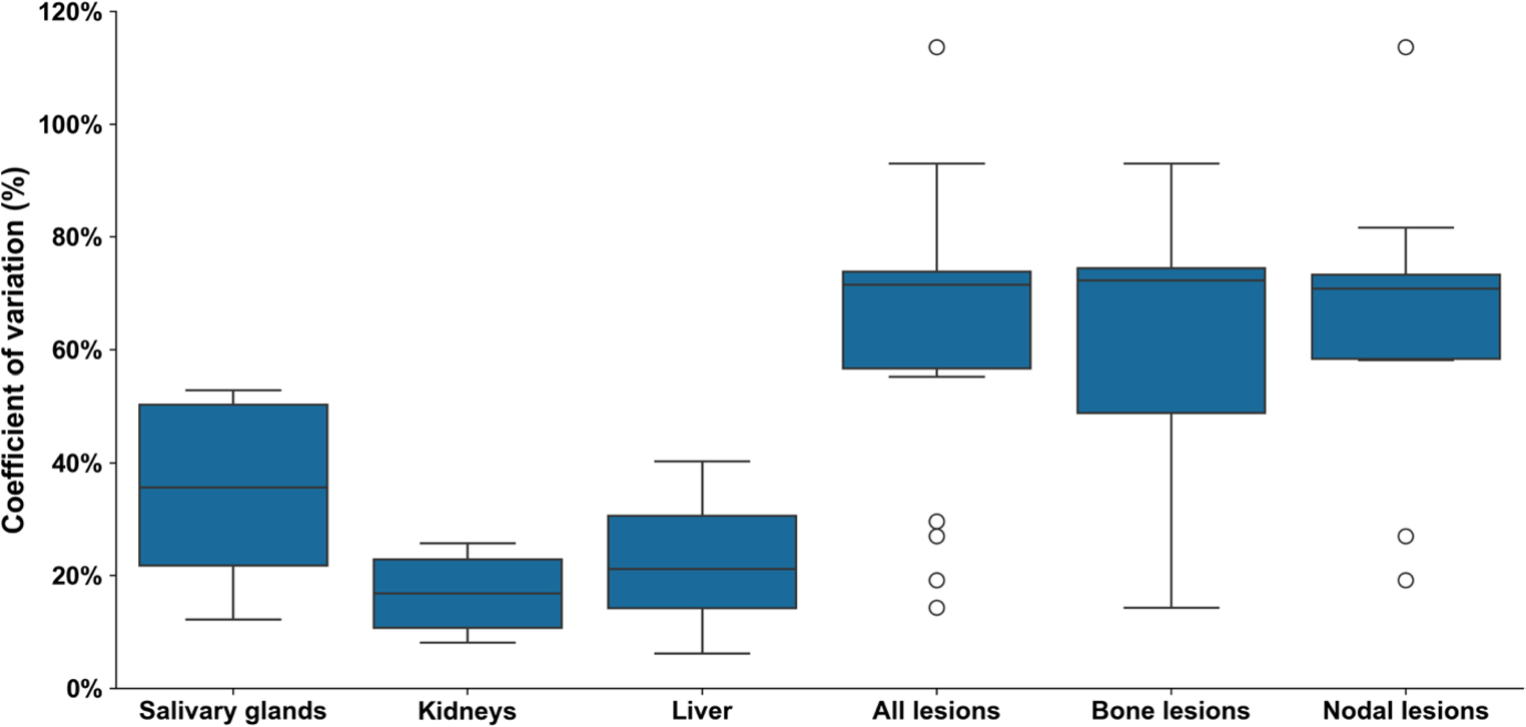
Box plot of coefficient of variation (CV) per structure for all patients and lesions. The CV is defined as the standard deviation expressed as a percentage of the mean and was calculated on a per patient/per lesion level

To further investigate the inter-center variability, the CV was determined for each organ or tumor at the patient level (Fig. 2, Supplementary Table 2). Among organs, the highest inter-center variation was seen for the salivary glands, with a median CV of 35.7% (range: 12.2%−52.9%). For kidneys and the liver, CVs were lower, at 16.8% (range: 8.1%−25.7%) and 21.2% (range: 6.2%−40.2%), respectively. The highest variation across centers was observed in the lesions, with a median CV of 71.5% (range: 14.3%−113.6%). There was no notable difference in CV between bone lesions and nodal lesions.

Salivary glands and in particular lesions were relatively small structures, with median volumes of 84 ml and 0.70 ml, respectively. The kidneys and liver were considerably larger, with median volumes of 346 ml and 1800 ml, respectively. Given that the inter-center variability (i.e., CV) was highest in the lesions, followed by the salivary glands, the correlation between CV and volume was investigated. For the salivary glands and liver, the CV showed a significant negative correlation with the average organ volume across all centers. Spearman’s ρ was − 0.72 (*p* = 0.019) for the salivary glands and − 0.70 (*p* = 0.025) for the liver (Supplementary Fig. 1 and Supplementary Table 3). This correlation of CV with the average volume across all centers was not present for kidneys and lesions.

### Variability due to volume definition for S-factors

**Fig. 3:**
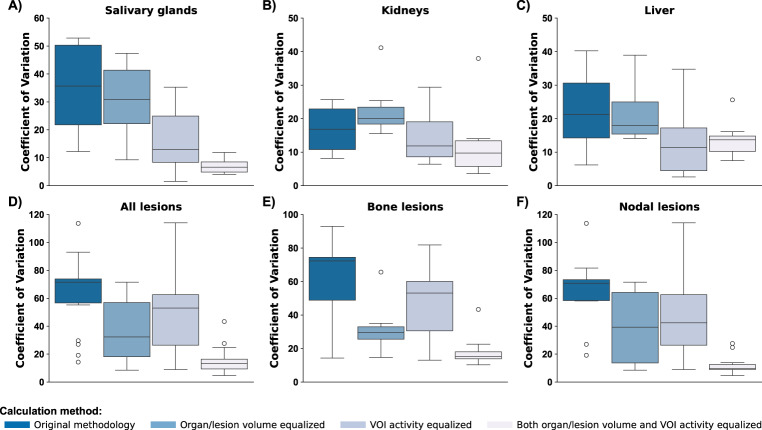
Impact of equalizing organ/lesion volumes, VOI activity and a combination on the coefficient of variation between centers for (**A**) kidneys, (**B**) liver, (**C**) salivary glands, (**D**) lesions, (**E**) bone lesions only, and (**F**) nodal lesions only

To assess the impact of individual steps within the dosimetry workflow on the inter-center variability, a series of homogenizations were performed across centers. Equalizing the volumes of organs and lesions for S-factors by using volumes determined by center 3, resulted in a reduction in median CV in all cases other than the kidneys (Fig. 3, Supplementary Table 2). In organs, the salivary glands and liver showed a decrease in median CV from 35.7% to 30.8% and from 21.2% to 18.0%, respectively (Fig. 3A and C). Conversely, the kidneys showed an increase in median CV from 16.8% to 20.1% after volume homogenization (Fig. 3B). The most pronounced decline in CV was observed for lesions, where the median CV decreased from 71.5% to 32.3% following volume homogenization (Fig. 3D). This decrease was more pronounced in bone lesions (Fig. 3E) compared to nodal lesions (Fig. 3F).

### Variability due to activity determination

To assess the impact of activity derived from delineated VOIs, the activity used as input for dosimetry calculations was homogenized across centers using activity determined by VOIs delineated by center 3. Fitting was then repeated using each center’s local clinical methodology. This homogenization resulted in a reduction in the median CV for all locations relative to the original methodology (Fig. 3, Supplementary Table 3). This effect was most pronounced in the salivary glands, where the median CV decreased from 35.7% to 12.9% (Fig. 3A). For the kidneys and the liver, this also resulted in a decrease in median CV, from 16.8% to 11.4% and from 21.2% to 11.5%, respectively (Fig. 3B and C). In the lesions, activity homogenization resulted in the median CV decreasing 71.5% to 53.0% respectively, following homogenization of activity input, this time the effect was more pronounced in nodal lesions compared to bone lesions (Fig. 3D, E and F).

### Variability due to remaining factors

When both volumes used for S-factor determination and activity were equalized concurrently, a reduction in CV between centers was observed. The largest decreases in median CV were observed in the lesions and salivary glands, with reductions in median CV from 71.5% to 13.1% and from 35.7% to 6.5% respectively. The kidneys and liver showed decreases from 16.8% to 9.7% and from 21.2% to 13.7%, respectively. The IQR of all organs and lesions, except for the kidneys, are also lowest after combining these homogenization steps (Fig. 3, Supplementary Table 2).

## Discussion

This study aimed to assess which factors have the biggest impact on inter-center variability in AD calculations by comparing AD outcomes and homogenizing dosimetry workflow steps using a patient dataset acquired at a single center. The data used was acquired at a single center, consisting of five timepoint SPECT/CT images from vertex to mid-thigh, representing an ideal dataset as input for dosimetry [25]. Despite the quality of the dataset and the centers’ experience, considerable inter-center variability is still present. Delineation of volumes was the biggest source of inter-center variability in AD. For organs, this was primarily due to VOI delineation for activity, whereas for lesions, it was largely driven by defining volumes used for S-factor determination. A major limitation of the study is that center 1 and center 3 collaborated previously on the determination of volumes for lesions, resulting in identical volumes used to determine S-factors. Meaning the variation in S-factor determination for lesions was primarily due to differences compared to center 3. In lesions, this could lead to an underestimation of some of the original variability due to the lack of independent lesion volume determination and a reduction of the volume-homogenizing effect. Given that the AD ground truth is inherently unknown due to data being derived from a patient dataset, definitive conclusions regarding the best approaches regarding dosimetry methodology cannot be drawn. Nevertheless, to derive meaningful dose–effect relationships, patient-specific organ volumes should be preferred when CT-based contouring is available [26]. In this study, Center 1 adopted ICRP reference organ masses to implement a simplified approach to facilitate dosimetry implementation in routine clinical practice.

To study inter-center variability, the CV was determined per patient on an organ and lesion level. The lesions showed the highest CV, median 71.5%, with no apparent differences between nodal or bone lesions. The large variability could be due to the patient cohort of oligometastatic patients, in which lesions are often small and more challenging to accurately delineate, corresponding to a high uncertainty in calculated AD. This is supported by the volume-homogenization effect, significantly reducing the CV to 32.3%. In addition to this, accurate quantification is challenging because of the low-resolution SPECT and partial volume effects. This effect is less important than the volume determination for S-factors, as the activity-homogenization effect only reduced the CV to 53.0%. The inter-center variability was substantially greater for lesions than organs, as illustrated by both the higher CV and by the maximum differences in reported AD between centers. A median maximum difference of 4.53 Gy/GBq may have a meaningful effect on the interpretation of dose-effect relations and clinical decision making on a patient level. While precise dose-effect relationships have not been established for radioligand therapy, reported mean tumor lesion doses are often in the ~ 3–12 Gy/GBq range [8], indicating that variability of the magnitude reported here could have considerable impact.

Similarly, the salivary glands, which are also relatively small and less easily identified on CT imaging, showed the highest median CV (35.7%) among the investigated organs. Even though the liver and kidneys are larger and thus more easily definable on imaging, the inter-center variability can still be problematic. For example, the observed median maximum inter-center difference in kidney AD was 0.20 Gy/GBq. When extrapolated to six cycles of 7.4 GBq (the current standard dosing scheme) this corresponds to a cumulative AD difference of approximately 8.9 Gy, assuming the dose remains constant across cycles. This difference represents nearly 40% of the current accepted renal dose limit of 23 Gy based on external beam radiotherapy [27]. Whilst it is acknowledged that the exact renal dose limit and its clinical implications for radionuclide therapies remains unknown, such differences highlight the importance of methodological consistency. These observations illustrate potential implications for patient management, particularly in context of potential future implementation of personalized dosimetry, as investigated in [^177^Lu]Lu-DOTATATE [10]. However, the present study focuses primarily on reproducibility rather than on predicting clinical outcomes.

All participating centers used an organ-based dosimetry approach and an identical input dataset. Therefore, the observed inter-center variability in the AD estimates were due to differences in their dosimetry methodology. Some of the observed variability may also reflect limitations of the organ-based dosimetry approach, which assumes uniform activity distribution, idealized organ and lesion geometry, and is susceptible to partial-volume effects as well as spill-in and spill-out effects, particularly in small lesions. Homogenization of the volumes used for S-factor determination had a large effect, particularly in the lesions. Across centers, various approaches were used to define these volumes. For instance, center 1 used standard masses defined in ICRP89 to determine S-values for organs. Whilst the advantage of using ICRP89 volumes is a standardized approach, they do not account for differences in patient anatomy, potentially contributing to the inter-center differences. This may explain why center 1 reported significantly lower salivary gland ADs than center 2 and 3, while salivary gland doses did not significantly differ between centers 2 and 3. However for larger organs, such as the kidneys and liver, the use of standard masses did not explain the difference as the liver AD differed significantly between center 3 and centers 1 and 2. Interestingly, equalizing the volume used to determine an S-value led to a small increase in median CV for the kidneys, from 16.8% to 20.1%. In contrast, for the lesions, this homogenization resulted in a large decrease of median CV. Given that the patients in this study were in oligometastatic hormone sensitive prostate cancer setting, many of the lesions were small metastases which makes determining an accurate volume more challenging. This is also evident from the number of excluded lesions, only 19 out of 41 lesions were included in the analysis. Lesions were predominantly excluded due to poor visibility or for being too small for accurate dosimetry on SPECT/CT, highlighting the challenges of lesion dosimetry in this patient cohort.

For organs, the largest source of variability was due to the delineation of VOIs to determine activity in each region: the activity-homogenization effect resulted in the largest decrease in median CV. This also led to a decrease in CV for lesions, although the activity-homogenization effect had a lesser impact on the variability in the lesions compared to organs. In this study, VOIs were delineated manually and thus for a large part center and operator dependent. In smaller lesions and harder to segment organs such as the salivary glands this can be due to partial volume effects and the resolution of SPECT relative to the organ/lesion size. It is striking that VOI delineation also played a large role in the CV between centers for larger organs such as the kidneys and liver. For example, the median CV decreased from 21.2% to 11.4% in the liver following homogenization of VOI activity. This in part may be due to the method chosen to draw a VOI based on CT-contours vs. the SPECT-signal and the method of background correction applied. The high impact of this step in the dosimetry workflow highlights the need for standardization of VOI delineation.

Although curve fitting approaches differed across centers, the residual inter-center variability homogenizing both volume and activity was markedly diminished. The remaining median CV in for example the salivary glands and lesions being 6.2% and 13.1%, respectively. This indicates that the difference in time-activity curve fitting method contributed less to the overall inter-center variability compared to volume determination for S-factors and VOI definition. Nonetheless, curve fitting is an important step in the dosimetry process. The use of population-based pharmacokinetic models and representative time-activity curve data can aid more consistent selection of fitting, particularly for organs-at-risk [28]. However, lesion kinetics often exhibit high variability both on a patient and lesion level, limiting the applicability of population-based fitting methods.

The SNMMI Dosimetry Challenge previously investigated factors contributing to the variability of dosimetry calculations [29–32]. In two [^177^Lu]Lu-DOTATATE datasets, CVs of 54.6% and 57.7% were reported for kidney dose, and 48.4% and 43.6% for liver dose across participating centers [32]. Lesions demonstrated even greater variability, with CVs in individual lesions ranging from 41.1% to 98.1%. In contrast, we observed lower median CVs of 16.8% and 21.2% for kidneys and liver, respectively, and a similarly high median CV of 71.5% for lesions. This underscores that lesions remain challenging for accurate dosimetry. The differences in reported CV may partly be attributed to a difference in radiotracers ([^177^Lu]Lu-DOTATATE versus [^177^Lu]Lu-PSMA-617), tumor type, localization and size, fewer and different SPECT/CT imaging timepoints, and the use of data from only two patients in the SNMMI challenge. Additionally, the larger number of participating centers and varying expertise in dosimetry calculations in the SNMMI study may have contributed to the increased variability. Similarly to our findings, the SNMMI dosimetry challenge showed that curve fitting is not a large source of variability, especially in healthy organs [31]. Instead, the highest variation stemmed from the VOI definition and the determination of volumes underlying S-factors. This is further supported by Finocchiaro and colleagues, who showed that the highest level of uncertainty in RLT are associated with these two factors [33]. In addition to inter-center variability, inter-user variability also contributes as VOI delineation and S-factor associated volume definition remain largely subjective in the absence of consensus guidelines, despite methodology being mostly center-dependent.

Standardized guidelines to delineate VOIs could reduce variability, like in external beam radiotherapy where guidelines were implemented and have shown to reduce inter-observer variability [34]. However, even with guidelines in place, some inter-observer variability will remain, as was shown in for example head and neck cancer radiotherapy [35]. Further standardization and reduction of variability can perhaps be achieved using artificial intelligence (AI)-based contouring of VOIs. Significant steps have been made using AI-based contouring, for example automatic bone marrow segmentation in [^177^Lu]Lu-PSMA-617 SPECT/CT and segmentation of the left ventricle in myocardial perfusion imaging [36, 37]. In addition, many dosimetry packages now include automatic CT-based segmentation (e.g. automatic kidney segmentation), reducing the reliance on observer- and center-specific variability. In addition, Monte Carlo based voxel-level dosimetry approaches have the potential to mitigate certain sources of variability inherent to the organ-based methods used here, particularly those arising from partial-volume effects and spill-in/spill-out phenomena. Nevertheless, voxel-level dosimetry remains sensitive to misalignment between sequential scans, which is especially challenging for small lesions and may introduce alternative sources of uncertainty. Future research should focus on AI-based approaches for regions with limited contrast to surrounding tissue, such as salivary glands and small lesions. With [^177^Lu]Lu-PSMA therapy now becoming more widely available, larger datasets are likely to become available for such model training in the foreseeable future.

## Conclusion

Substantial inter-center variability in ADs was observed in this study. The largest factors for this variability were activity delineation for organs, and the volumes associated with the S-factors for lesions. This underscores the need for standardized delineation within the dosimetry workflow and for a broadly supported inter-center consensus to ensure reproducible and comparable dosimetry results.

## Supplementary Information

Below is the link to the electronic supplementary material.

ESM 1 (DOCX 304 KB)

## Data Availability

The datasets generated during and/or analyzed during the current study are available from the corresponding author on reasonable request.
