# Supplementary material for: Inter-center variability of dosimetry methodology and the impact on reported absorbed dose for [177Lu]Lu-PSMA-617 therapy
Source: Eur J Nucl Med Mol Imaging. 2026 Mar 26;53(9):5384–94. doi: 10.1007/s00259-026-07832-3 (PMC13315099; doi:10.1007/s00259-026-07832-3)
Supplement: Supplementary file 1 — (DOCX 304 KB) [file 259_2026_7832_MOESM1_ESM.docx]

**Supplementary Information**

**Materials S1: Acquisition and reconstruction parameters**

**PET-imaging acquisition and reconstruction**

Acquisition time was 4 minutes per bed-position for the pelvis and 3 minutes for the remaining bed positions. Image reconstruction was performed using OSEM-TOF with 6 iterations and 12 subsets. Matrix size was 440 with a 4 mm Gaussian filter applied. Attenuation correction was performed using low-dose CT imaging performed using standard clinical parameters of 110 kV and 48 mA. Diagnostic CT images were reconstructed iteratively using a B31f-convolution kernel with a 780 mm field of view.

**SPECT-imaging acquisition and reconstruction**

For SPECT/CT imaging a Siemens Symbia T16 or Intevo Bold gamma camera was used with a 128 × 128 matrix over 3 bed positions at each time point (including head/neck, abdomen, and pelvis region). The energy window was set at 20% around 208 keV with a lower scatter window of 20% around 170 keV. SPECT reconstruction was performed using an OSEM algorithm with Flash 3D and resolution recovery, employing 4 iterations and 8 subsets. A Gaussian post-filter with a kernel size of 4 mm was applied. The reconstructed voxel size was 4.8 × 4.8 × 4.8 mm. Scatter correction was performed using the dual-energy window method, and attenuation correction was based on the corresponding CT data.

**Supplementary table 1:** List of lesions identified for each patient with reasons for exclusion for each center.

| **Patient #** | **Lesion #** | **Center 1** | **Center 2** | **Center 3** | **Included in analysis?** |
| --- | --- | --- | --- | --- | --- |
| **1** | **1** | **Included** | **Accurate time-activity curve fit not possible** | **Included** | **No** |
| **2** | **1** | **Included** | **Included** | **Included** | **Yes** |
|  | **2** | **Included** | **Included** | **Included** | **Yes** |
|  | **3** | **Included** | **Included** | **Included** | **Yes** |
|  | **4** | **Lesion too small for dosimetry** | | | **No** |
| **3** | **1** | **Included** | **Included** | **Included** | **Yes** |
|  | **2** | **Not measurable** | | | **No** |
|  | **3** | **Not measurable** | | | **No** |
|  | **4** | **Not measurable** | | | **No** |
| **4** | **1** | **Included** | **Included** | **Included** | **Yes** |
|  | **2** | **Included** | **Included** | **Included** | **Yes** |
|  | **3** | **Lesion too small for dosimetry** | | | **No** |
|  | **4** | **Included** | **Included** | **Included** | **Yes** |
|  | **5** | **Included** | **Accurate time-activity curve fit not possible** | **Included** | **No** |
|  | **6** | **Included** | **Included** | **Included** | **Yes** |
|  | **7** | **Included** | **Included** | **Included** | **Yes** |
|  | **8** | **Lesion too small for dosimetry** | | | **No** |
|  | **9** | **Included** | **Included** | **Included** | **Yes** |
| **5** | **1** | **Included** | **Included** | **Included** | **Yes** |
|  | **2** | **Included** | **Accurate time-activity curve fit not possible** | **Poor visibility of lesion on early timepoints – accurate time-activity curve fit not possible** | **No** |
|  | **3** | **Included** | **Included** | **Included** | **Yes** |
|  | **4** | **Included** | **Included** | **Poor visibility of lesion on early timepoints – accurate time-activity curve fit not possible** | **No** |
|  | **5** | **Included** | **Included** | **Included** | **Yes** |
|  | **6** | **Lesion too small for dosimetry** | | | **No** |
| **6** | **1** | **Included** | **Included** | **Included** | **Yes** |
|  | **2** | **Included** | **Included** | **Included** | **Yes** |
|  | **3** | **Included** | **Included** | **Not observed** | **No** |
|  | **4** | **Lesion too small for dosimetry** | | | **No** |
| **7** | **1** | **Included** | **Included** | **Included** | **Yes** |
|  | **2** | **Included** | **Included** | **Included** | **Yes** |
|  | **3** | **Lesion too small for dosimetry** | | | **No** |
| **8** | **1** | **Lesion too small for dosimetry** | | | **No** |
|  | **2** | **Included** | **Accurate time-activity curve fit not possible** | **Included** | **No** |
|  | **3** | **Lesion too small for dosimetry** | | | **No** |
| **9** | **1** | **Included** | **Included** | **Included** | **Yes** |
|  | **2** | **Included** | **Accurate time-activity curve fit not possible** | **Included** | **No** |
|  | **3** | **Included** | **Included** | **Not observed** | **No** |
|  | **4** | **Lesion too small for dosimetry** | | | **No** |
| **10** | **1** | **Included** | **Included** | **Included** | **Yes** |
|  | **2** | **Included** | **Included** | **Not observed** | **No** |
|  | **3** | **Included** | **Included** | **Not observed** | **No** |


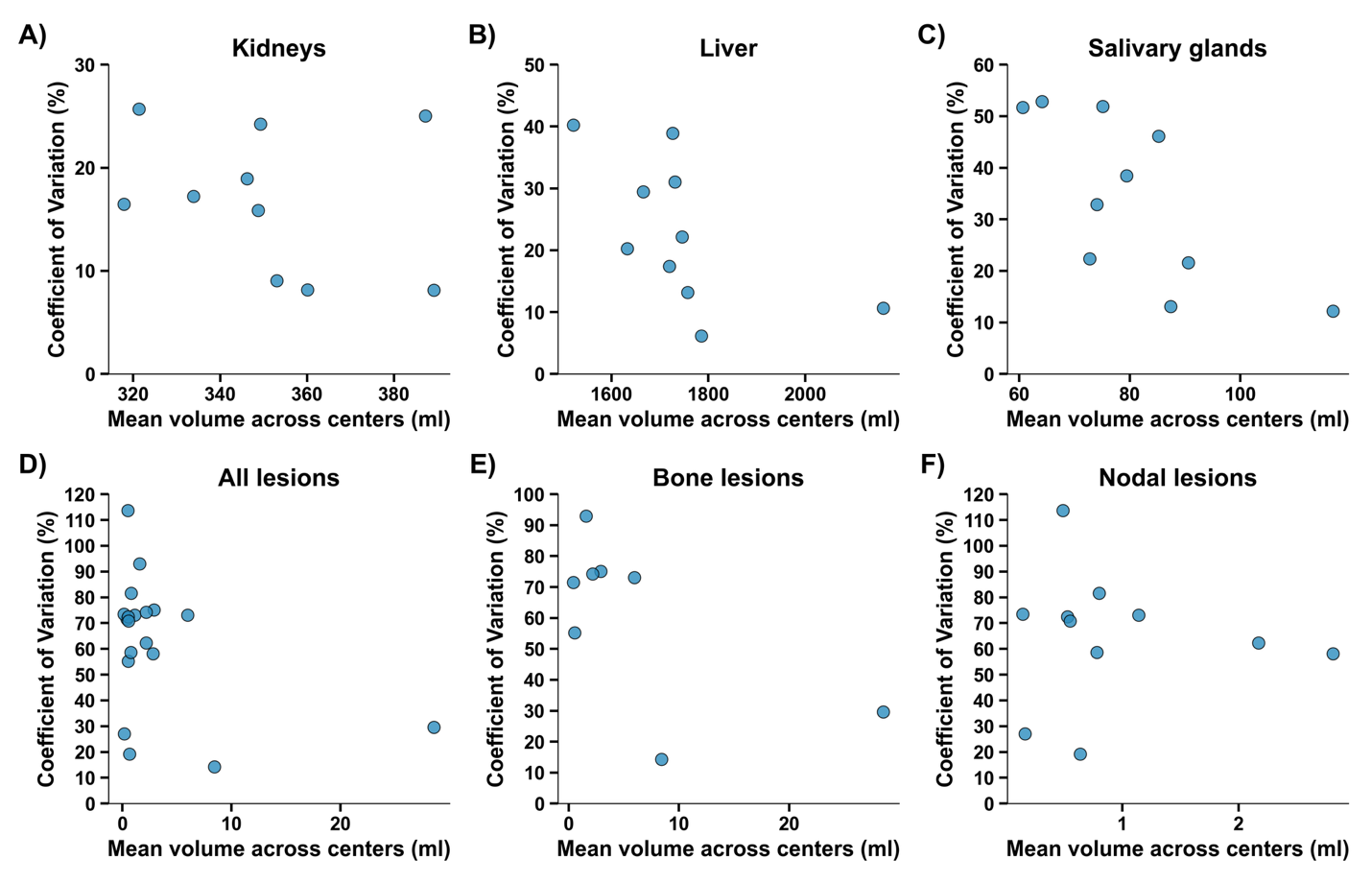


**Supplementary figure 1:** Scatterplot of the coefficient of variation, determined by each center's original methodology, against the mean volume across all centers for each location.

**Supplementary table 2:** Median CV (range: min-max) for each structure and each methodology.

| **Structure** | **Original methodology** | **Organ/lesion volume equalized** | **VOI activity equalized** | **Both organ/lesion volume and VOI activity equalized** |
| --- | --- | --- | --- | --- |
| **Salivary glands** | 35.7% (range: 12.2%-52.9%) | 30.8% (range: 9.3%-47.4%) | 12.9% (range: 1.5%-35.3%) | 6.6% (range: 4.0%-11.9%) |
| **Kidneys** | 16.8% (range: 8.1%-25.7%) | 20.1% (range: 15.5%-41.2%) | 11.8% (range: 6.4%-29.4%) | 9.7% (range: 3.6%-38.0%) |
| **Liver** | 21.2% (range: 6.2%-40.2%) | 18.0% (range: 14.1%-39.0%) | 11.4% (range: 2.6%-34.7%) | 13.7% (range: 7.4%-25.6%) |
| **All lesions** | 71.5% (range: 14.3%-113.6%) | 32.3% (range: 8.5%-71.5%) | 53.0% (range: 8.9%-114.0%) | 13.1% (range: 4.7%-43.3%) |
| **Bone lesions** | 72.3% (range: 14.3%-93.0%) | 29.7% (range: 14.7%-65.6%) | 53.1% (range: 13.0%-81.9%) | 15.2% (range: 10.3%-43.3%) |
| **Nodal lesions** | 70.8% (range: 19.2%-113.6%) | 39.2% (range: 8.5%-71.5%) | 42.4% (range: 8.9%-114.0%) | 9.6% (range: 4.7%-27.6%) |

**Supplementary table 3:** Spearman’s correlation between mean volume and CV (%) per location

| **Structure** | **Spearman ρ** | **p-value** |
| --- | --- | --- |
| **Kidneys** | -0.43 | 0.214 |
| **Salivary glands** | -0.72 | 0.019 |
| **Liver** | -0.70 | 0.025 |
| **All lesions** | -0.12 | 0.632 |
| **Bone lesions** | -0.38 | 0.352 |
| **Nodal lesions** | -0.21 | 0.537 |
